# High-Resolution Melting PCR-Based SNP Germline Genotyping of *KLK3*, *JAZF1*, and *HNF1B* in Prostate Cancer and Benign Prostatic Hyperplasia in Nigerian Men—A Pilot Study

**DOI:** 10.3390/diagnostics16162521

**Published:** 2026-08-10

**Authors:** Lateef Adegboyega Sulaimon, Ayorinde Babatunde James, Aishat Abisola Akinbami, Oladayo Musa Babalola, Grace Folashayo Oni, Ganiu Adigun Idris, Mayowa Agbi, Akintayo Ashraf Akintola, Modinat Olawunmi Lawal, Adewale Segun James

**Affiliations:** 1 Department of Biochemistry, Faculty of Life Science, University of Abuja, Abuja 900109, Nigeria; 2 The GeneLab Bioscience, Yaba, Lagos 101212, Nigeria; 3 Department of Chemical Sciences, College of Natural and Applied Science, Crescent University, Abeokuta 111105, Nigeria; 4 Department of Biochemistry, Faculty of Life Science, University of Lagos, Lagos 101017, Nigeria; 5 Department of Biology, Teachers College and Institute for Phylogenomics and Evolution, Kyungpook National University, Daegu 41566, Republic of Korea; 6 School of Integrated Biomedical Sciences, USF Morsani College of Medicine, University of South Florida, Tampa, FL 33620, USA

**Keywords:** prostate cancer, benign prostatic hyperplasia, high-resolution melting, SNP genotyping, *KLK3*, *HNF1B*, *JAZF1*, molecular diagnostics

## Abstract

**Background/Objectives**: Prostate cancer (PCa) and benign prostatic hyperplasia (BPH) are highly prevalent among African men. While single-nucleotide polymorphisms (SNPs) in *KLK3, HNF1B*, and *JAZF1* are established risk markers in Western cohorts, their specific allele frequencies and diagnostic utility remain poorly characterized in African populations. This study evaluated the diagnostic accuracy, sensitivity, and cost-effectiveness of High-Resolution Melting (HRM) PCR for germline SNP genotyping in a cohort of Nigerian men. **Methods:** Peripheral blood buffy coat DNA was extracted from 31 male participants attending the urology clinic at Lagos University Teaching Hospital, classified into PCa (*n* = 13), BPH (*n* = 12), and healthy controls (*n* = 6). Real-time PCR and HRM analysis were optimized to genotype three specific risk variants: rs2735839 *(KLK3)*, rs4430796 *(HNF1B)*, and rs10486567 *(JAZF1)*. Allelic discrimination was validated using sequence-verified, synthetic positive controls for homozygous wild-type and mutant variants. HRM analysis generated reproducible amplification and melting profiles that enabled clear discrimination of wild-type, heterozygous, and variant genotypes for all three loci. **Results:** The risk-associated alleles of *KLK3* (G), *HNF1B* (C), and *JAZF1* (T) occurred more frequently among patients with prostate cancer than among those with benign prostatic hyperplasia. Clinically, prostate cancer patients also presented with markedly higher PSA concentrations, older age at diagnosis, and higher Gleason scores, consistent with more aggressive disease. The optimized HRM workflow required less than two hours from amplification to genotype assignment and eliminated the need for post-PCR processing, making it suitable for routine molecular testing in resource-constrained laboratories. **Conclusions:** HRM-based genotyping of *KLK3* (rs2735839), *HNF1B* (rs4430796), and *JAZF1* (rs10486567) provides a highly reproducible, rapid, and low-cost molecular tool for mapping prostate disease risk variants. Because it requires no post-PCR processing, this closed-tube HRM workflow is ideally suited for resource-limited clinical settings to enhance early risk stratification and differentiate malignant PCa from benign BPH.

## 1. Introduction

Prostate cancer (PCa) and benign prostatic hyperplasia (BPH) are among the most prevalent urological disorders affecting aging men worldwide. Their incidence increases markedly with advancing age, and both conditions contribute substantially to morbidity and healthcare burden. In sub-Saharan Africa, late presentation and limited access to advanced diagnostic facilities remain common, resulting in poor clinical outcomes, particularly for prostate cancer patients [1,2]. In Nigeria, prostate cancer is frequently diagnosed at advanced stages, with correspondingly high mortality rates [3].

Current screening and diagnostic approaches for prostate diseases, including prostate-specific antigen (PSA) testing and digital rectal examination (DRE), are widely used but lack sufficient specificity to reliably differentiate malignant prostate cancer from benign prostatic enlargement [4]. Elevated PSA levels may be observed in both PCa and BPH, leading to diagnostic uncertainty, unnecessary biopsies, delayed treatment, and increased patient anxiety. These limitations underscore the need for more accurate and accessible molecular diagnostic tools that can improve early detection and risk stratification, particularly in resource-limited settings.

Prostate cancer is a genetically complex and polygenic disease influenced by both inherited and acquired genetic variations [5]. Genome-wide association studies (GWAS) have identified multiple single-nucleotide polymorphisms (SNPs) associated with prostate cancer susceptibility and progression [6]. Among these, variants in *KLK3*, *HNF1B*, and *JAZF1* have been consistently linked to prostate cancer risk across diverse populations [7,8]. The *KLK3* gene encodes prostate-specific antigen, a central biomarker in the diagnosis of prostate disease, while *HNF1B* functions as a transcription factor involved in cellular differentiation and tumorigenesis [9]. However, data on the clinical relevance of these variants in African populations remain limited, despite evidence of more aggressive disease phenotypes and higher mortality rates [10]. Genetic variation within the JAZF1 (Juxtaposed with Another Zinc Finger Protein 1) locus, particularly the intronic single-nucleotide polymorphism rs10486567 on chromosome 7p15.2, has been consistently implicated in prostate cancer susceptibility through genome-wide association studies (GWAS). Initial large-scale analyses identified rs10486567 as significantly associated with prostate cancer risk, achieving genome-wide significance (*p* ≈ 7.8 × 10^−11^) and conferring modest increases in risk (OR ~1.19 for heterozygotes and ~1.37 for homozygotes) [11].

High-resolution melting (HRM) analysis is a rapid, sensitive, and cost-effective post–polymerase chain reaction (PCR) technique for detecting sequence variations such as SNPs [12]. HRM enables discrimination of genotypes based on differences in DNA melting behavior, without the need for labeled probes or sequencing, making it particularly suitable for large-scale screening and for laboratories with limited resources. When combined with conventional PCR, HRM offers a practical and reliable approach for molecular profiling of clinically relevant genetic variants.

The clinical implementation of HRM-based SNP genotyping offers a practical opportunity to integrate inherited genetic information into routine prostate cancer evaluation. By combining conventional clinical parameters with molecular susceptibility markers, HRM may improve diagnostic confidence, facilitate patient stratification, and support more personalized approaches to prostate disease management without substantially increasing laboratory costs.

Against this background, the present pilot study evaluated the feasibility of high-resolution melting PCR for genotyping three clinically relevant prostate cancer susceptibility polymorphisms—*KLK3* (rs2735839), *HNF1B* (rs4430796), and *JAZF1* (rs10486567)—in a cohort of Nigerian men with prostate cancer, benign prostatic hyperplasia, and healthy controls. We further assessed the potential of HRM-based genotyping as a rapid, reproducible, and affordable molecular diagnostic approach that could complement existing clinical tools for prostate disease evaluation in resource-limited healthcare settings.

## 2. Materials and Methods

### 2.1. Ethical Approval

This was a hospital-based, cross-sectional molecular study conducted at Lagos University Teaching Hospital (LUTH), Lagos, Nigeria. Ethical approval was obtained on 10 November 2025, from the Health Research Ethics Committee (HREC) of the College of Medicine, University of Lagos (Approval No: CMUL/HREC/09/25/2132). All study procedures were conducted in accordance with the principles of the Declaration of Helsinki.

### 2.2. Chemicals and Reagents

Genomic DNA was extracted using the QIAamp^®^ DNA Mini Kit (Qiagen GmbH, Hilden, Germany). Other reagents included nuclease-free water, absolute ethanol, ELISA kits for prostate-specific antigen (PSA) determination (Sigma-Aldrich, St. Louis, MO, USA), HRM qPCR Master mix, Qubit™ dsDNA Broad Range Assay reagents, and a DNA ladder (Thermo Fisher Scientific, Waltham, MA, USA). HPLC-purified primers and gene fragments were synthesized by TGL, Lagos, Nigeria.

### 2.3. Study Population

Participants were consecutively recruited from the urology clinic of Lagos University Teaching Hospital (LUTH), Lagos, Nigeria. The study objectives, procedures, potential risks, and participants’ rights, including the right to withdraw at any time, were clearly explained before consent was obtained. A total of 31 adult male participants aged 51–80 years were included in the final analysis and categorized into three groups: prostate cancer patients (PCa; *n* = 13), benign prostatic hyperplasia patients (BPH; *n* = 12), and apparently healthy controls (*n* = 6).

Diagnosis of PCa and BPH was based on clinical evaluation, prostate-specific antigen (PSA) levels, histopathological findings, and urological assessment where applicable. Benign prostatic hyperplasia was diagnosed using clinical examination, ultrasonography where indicated, PSA measurement, and exclusion of malignancy.

Age-matched apparently healthy men with no previous diagnosis of prostate disease, malignancy, or chronic inflammatory disorders served as the reference population.

Sociodemographic and clinical data, including age, ethnicity, body mass index (BMI), blood pressure, PSA levels, Gleason scores, lifestyle factors, and relevant medical history, were collected using structured questionnaires and medical records. The questionnaire is provided as a Supplementary File S1.

#### Inclusion and Exclusion Criteria

Participants were eligible if they were at least 50 years of age; provided written informed consent; and had a confirmed diagnosis of prostate cancer or benign prostatic hyperplasia, or were apparently healthy controls without evidence of prostate disease.

Individuals were excluded if they declined participation; had insufficient blood samples for molecular analysis; had degraded DNA samples that failed quality-control assessment; and had incomplete clinical records required for the study.

### 2.4. Genomic DNA Extraction and Quantification

Genomic DNA from all PCa, BPH, and healthy control samples was extracted from buffy coat samples using the same QIAamp DNA Mini Kit (Qiagen, Hilden, Germany) according to the manufacturer’s instructions. Briefly, buffy coat aliquots were lysed in the presence of Proteinase K and Buffer AL, followed by ethanol precipitation. The lysates were applied to QIAamp silica membrane spin columns, washed sequentially with Buffers AW1 and AW2, and genomic DNA was eluted in Buffer AE according to the manufacturer’s instructions. DNA purity was assessed by absorbance spectrophotometry using a NanoDrop One Spectrophotometer (Thermo Fisher Scientific, USA), and DNA concentration was quantified fluorometrically using the Qubit 4 Fluorometer in conjunction with the Qubit dsDNA Broad Range (BR) Assay Kit (Thermo Fisher Scientific, USA). Samples meeting quality control requirements were used for downstream HRM-PCR applications [13].

To minimise variation in amplification and melting profiles, DNA samples were quantified (Table A1) before dilution and were adjusted to comparable working concentrations. Dilutions were prepared using nuclease-free water and thoroughly mixed before aliquoting into the reaction wells. Samples with inadequate purity or concentration were excluded from downstream HRM analysis. Particular attention was given to consistent template input because differences in DNA concentration may influence fluorescence intensity, melting-curve alignment, and genotype clustering in normalized and difference plots.

### 2.5. Measurement of Prostate-Specific Antigen (PSA)

The prostate-specific antigen levels were measured using ELISA Kits from Sigma-Aldrich according to the manufacturer’s protocol [14]. After the serum was separated from the collected blood samples, a wash buffer was also prepared. The desired number of coated strips was placed into the holder. A PSA standard control (25 µL) and the patient’s sera were pipetted. Anti-PSA conjugate reagent (100 µL) was added to each well and was incubated for 60 min at room temperature (18–26 °C). The liquid was removed from all wells, and the wells were washed three times with 300 µL of 1x Wash Buffer, after which the wells were blotted dry on absorbent paper. TMB substrate (100 µL) was added to each well, followed by incubation at room temperature for 15 min. Subsequently, 50 µL of Stop Solution was added to each well, and the absorbance was measured at 450 nm using an ELISA reader within 15 min of adding the Stop Solution. PSA concentrations were then determined from the standard calibration curve.

### 2.6. Selection of SNPs and Primer Design

Three prostate cancer-associated single nucleotide polymorphisms (SNPs), rs2735839 in the *KLK3* gene, rs4430796 in the *HNF1B* gene, and rs10486567 in the *JAZF1* gene, were selected based on their established associations with prostate cancer susceptibility identified through genome-wide association studies (GWAS) (Table 1). Primers flanking each target SNP region (Table 2) were designed using the NCBI Primer-BLAST tool to ensure specificity, appropriate melting temperature (Tm), optimal GC content, and amplicon lengths compatible with High-Resolution Melting (HRM) analysis. Synthetic DNA controls representing each allelic variant of the selected SNPs were designed and synthesized, and subsequently used as positive controls to validate genotyping accuracy and facilitate the interpretation of HRM melting profiles.

### 2.7. HRM-PCR and Melting Analysis

Real-time PCR and HRM analyses were performed using QuantStudio 5 Real-Time PCR System (Applied Biosystems, Waltham, MA, USA). Each reaction mixture (18 µL) contained 0.2 µL of DNA template or synthetic positive controls for the different alleles across the three genes (*KLK3*, *HNF1B* and *JAZF1*), 4 µL of HRM SYBR Green Master Mix, 0.4 µL of gene-specific Forward primer, 0.4 µL of gene-specific Reverse primer, and 13 µL of nuclease-free water. Negative control reactions contained nuclease-free water in place of DNA template. PCR amplification was initiated with an enzyme activation and initial denaturation step at 95 °C for 10 min, followed by 40 cycles of denaturation at 95 °C for 15 s, annealing at 59 °C for 20 s, and extension at 72 °C for 20 s. HRM analysis was subsequently performed following a conditioning step at 95 °C for 10 s, with melting from 60 °C to 95 °C at a ramp rate of 0.3 °C/s. Melting curves were analyzed to differentiate genotypes based on their characteristic melting profiles using hrmpy (v0.1), an open-source Python package for High-resolution melting analysis [15,16,17].

#### HRM Assay Optimisation and Troubleshooting

Each HRM assay was optimised separately because melting behaviour is influenced by amplicon length, GC content, primer specificity, DNA concentration, reaction composition, and instrument settings. Primer pairs were initially evaluated for specific amplification and the absence of non-specific products. Annealing conditions were adjusted to obtain a single, well-defined amplification product and reproducible melt profiles.

DNA input was also systematically assessed because variation in template concentration can alter amplification efficiency, fluorescence intensity, curve alignment, and genotype clustering. Initial reactions conducted using relatively concentrated DNA produced variable fluorescence intensities and less distinct grouping in the normalized and difference plots. Consequently, DNA samples were diluted and reassessed. Dilutions of 1:100 and 1:1000 produced more consistent amplification profiles and improved separation of genotype clusters.

Synthetic DNA fragments representing the relevant alleles were used as genotype controls and as references for interpretation of the melt curves. Pre-melt and post-melt normalization regions were selected outside the principal transition zone, and the wild-type control was used as the reference in the difference plots. Assay acceptance was based on specific amplification, reproducible melting behaviour, distinct genotype clustering, and absence of amplification in the no-template control.

All clinical samples, synthetic genotype controls, and no-template controls were analysed in duplicate. Genotype assignments were accepted only when both replicate reactions generated concordant amplification curves, normalized melting profiles, and difference plots. Samples with discordant replicate profiles were repeated.

### 2.8. Statistical Analysis

Statistical analyses were performed using SPSS version 27.0.1 and GraphPad Prism version 11.0.1. Continuous variables were expressed as median (interquartile range) or mean ± standard deviation as appropriate. Categorical variables were summarized as frequencies and percentages.

Genotype distributions between prostate cancer and BPH groups were compared using Fisher’s exact test because of the small sample size. Allelic associations were estimated by calculating odds ratios (ORs) with 95% confidence intervals (95% CI). Hardy–Weinberg equilibrium (HWE) was assessed in the non-cancer group using the exact test. Owing to the exploratory nature of this pilot study and limited sample size, statistical power was estimated post hoc. Statistical significance was defined as *p* < 0.05.

## 3. Results

### 3.1. Sociodemographic Characteristics of Study Participants

**Figure 1:**
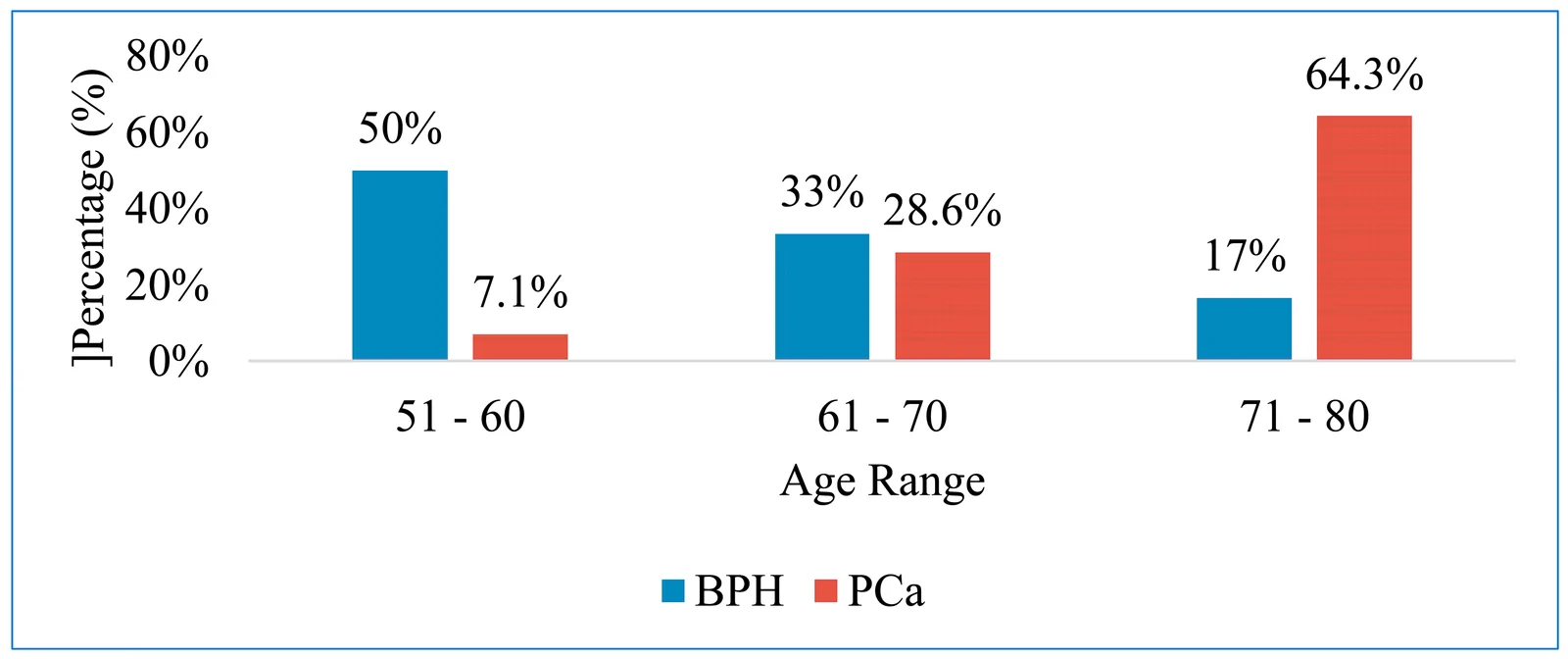
The age range distribution of Benign Prostatic Hyperplasia (BPH) and Prostate Cancer (PCa) patients. Data show BPH being more common in men aged 61–70, while PCa incidence peaks in men aged 71–80. BPH: Benign Prostatic Hyperplasia. PCa: Prostate Cancer.

The age distribution of participants with benign prostatic hyperplasia (BPH) and prostate cancer (PCa) is presented in Table 3 and Figure 1. BPH was most prevalent among men aged 61–70 years (50%), whereas prostate cancer incidence peaked in the 71–80-year age group (64.3%).

Total PSA concentrations were significantly higher in patients with prostate cancer group than in those with benign prostate hyperplasia (*p* = 0.022).

Educational attainment among participants is shown in Table 3. Approximately half of the study population had completed primary education, with a higher proportion of PCa patients in this category, while the remaining participants had attained tertiary education.

### 3.2. Clinical History and Characteristics

A comparison of key clinical parameters between BPH and PCa patients is presented in Table 4. Prostate cancer patients exhibited higher mean body mass index (25.09 ± 0.12 kg/m^2^), higher systolic blood pressure (143 ± 9.35 mmHg), and markedly elevated PSA levels (79.45 ± 0.23 ng/mL) compared with BPH patients (BMI: 22.48 ± 0.08 kg/m^2^; systolic BP: 130 ± 10 mmHg; PSA: 9.16 ± 0.12 ng/mL).

**Figure 2:**
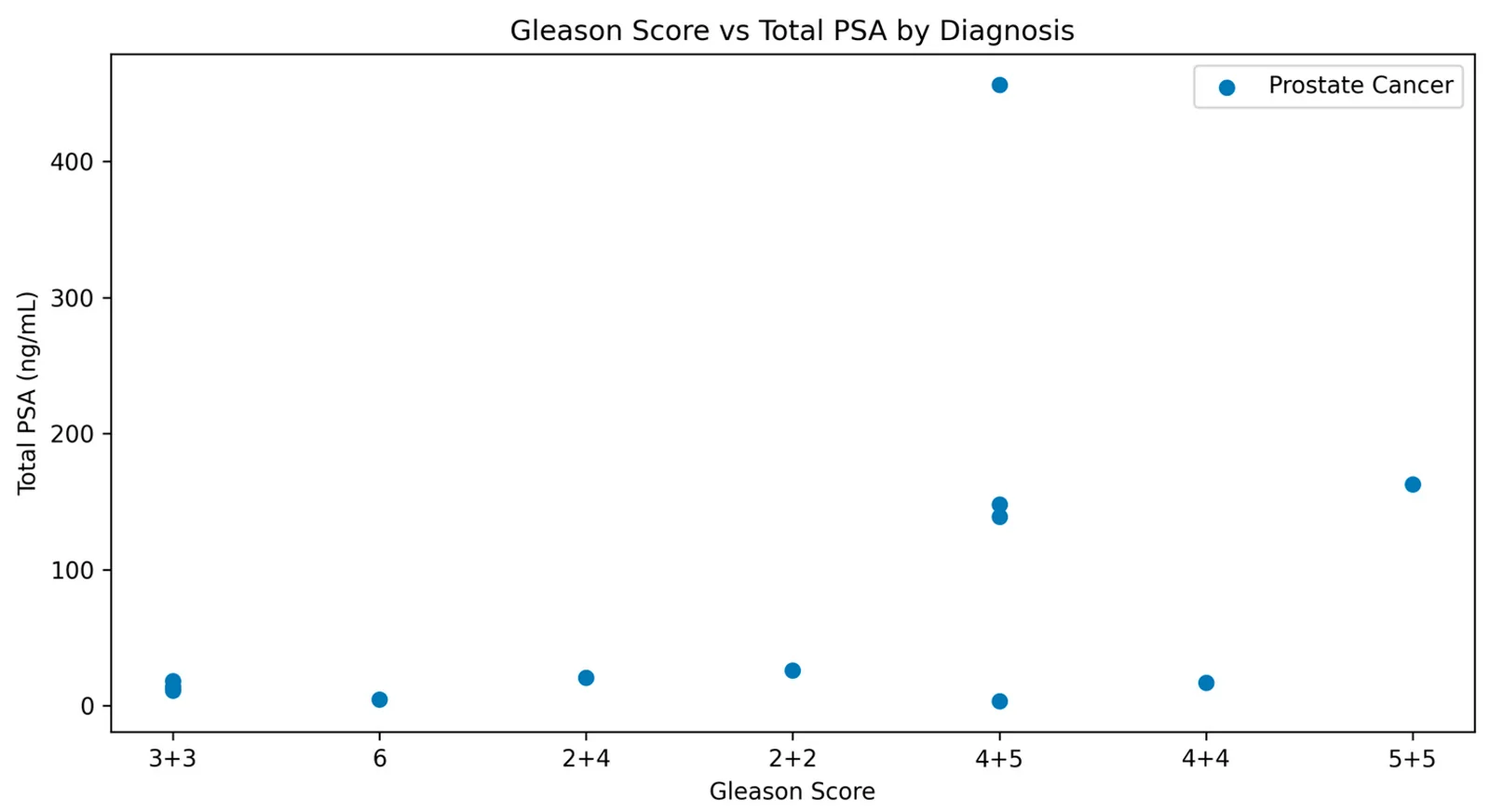
Relationship between Gleason score and total PSA concentration among patients with prostate cancer. Each point represents an individual patient. PSA levels generally increased with tumor grade, with the highest concentrations observed in patients with Gleason scores 4+5 and 5+5. Substantial variability in PSA values was observed within Gleason score categories.

Gleason score distribution among prostate cancer patients is shown in Figure 2. Most PCa patients presented with high-grade tumors, with Gleason scores of 4+4, 4+5, or 5+5, indicating aggressive disease.

**Figure 3:**
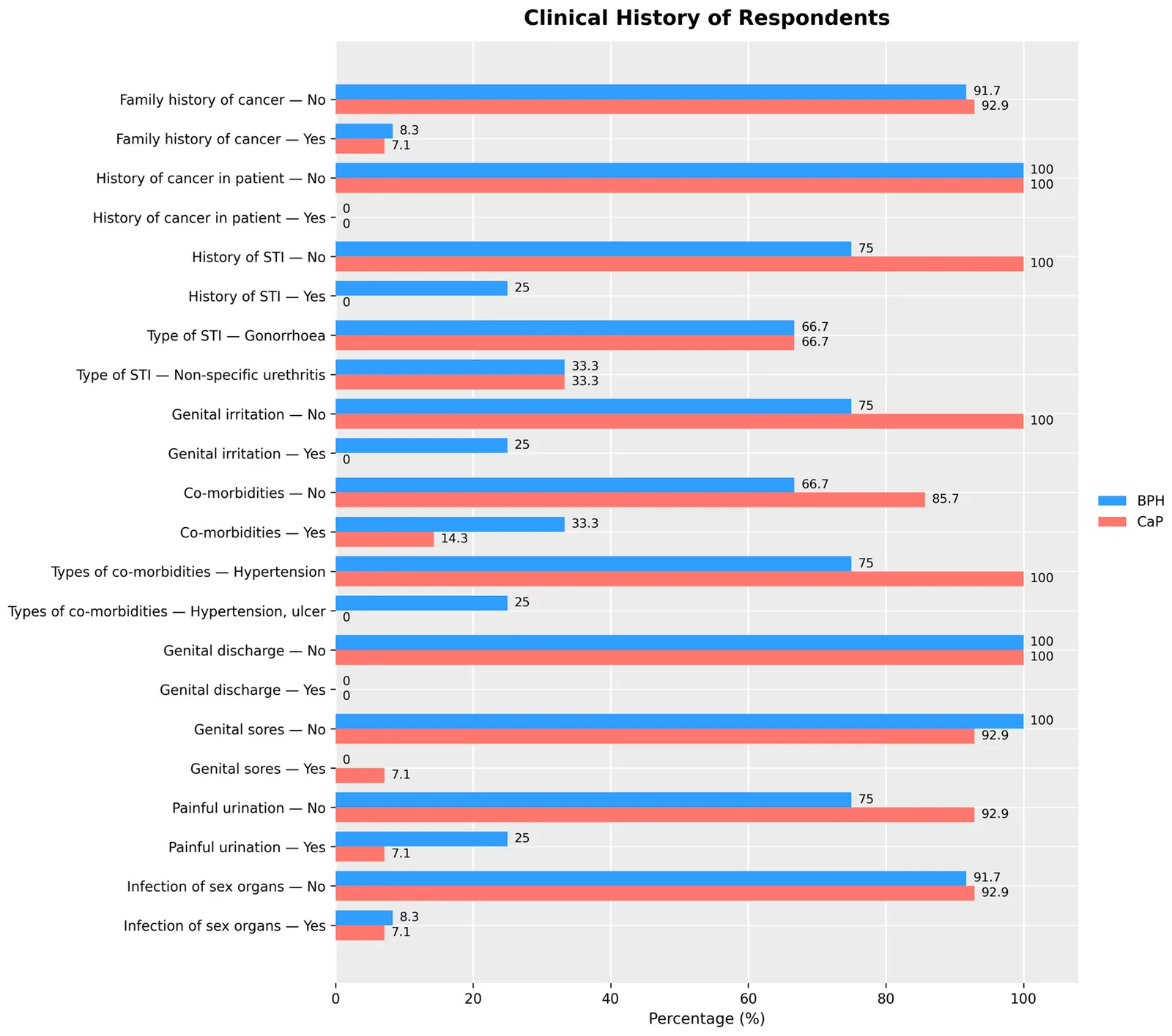
Clinical characteristics of participants with benign prostatic hyperplasia (BPH) and prostate cancer (PCa). Data are presented as percentages within each diagnostic group.

As shown in Figure 3 below, most participants in both groups reported no family history of cancer (BPH: 91.7%; PCa: 92.9%), and none reported a previous cancer diagnosis. A history of sexually transmitted infection (STI) was observed only among BPH patients (25.0%), with gonorrhea accounting for 66.7% of STI cases and non-specific urethritis accounting for 33.3%. Genital irritation and painful urination were more frequently reported among BPH patients (25.0% each) than among PCa patients (0% and 7.1%, respectively). Co-morbidities were more common in the BPH group (33.3%) compared with the PCa group (14.3%), with hypertension being the predominant co-morbidity in both groups. Genital discharge was not reported in either group, whereas genital sores were reported only among PCa patients (7.1%). Infection of the sex organs was uncommon and occurred at similar frequencies in both groups (BPH: 8.3%; PCa: 7.1%).

### 3.3. Real-Time PCR and High-Resolution Melting Analysis

**Figure 4:**
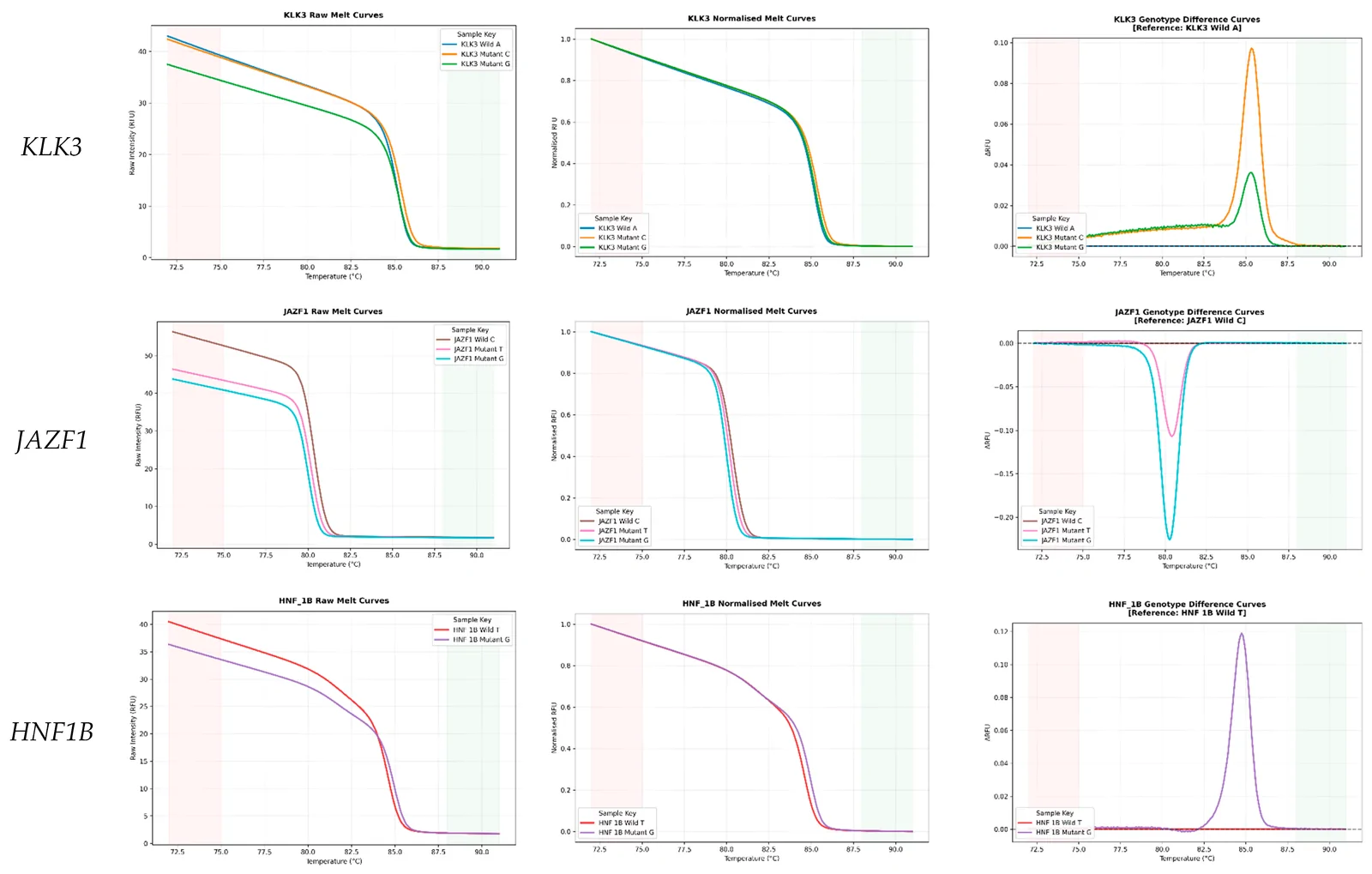
High-resolution melting (HRM) analysis of *KLK3*, *JAZF1*, and *HNF1B*. Raw melt curves, normalized melt curves, and genotype difference plots are shown for wild-type (WT) and mutant (MT) samples. Difference plots were generated using the WT genotype as the reference, demonstrating distinct melting profiles that allow clear discrimination between wild-type and mutant variants. Pre-melt and post-melt normalization regions are shaded in pink and green, respectively.

Initial real-time PCR amplification and HRM analysis of synthetic positive controls demonstrated clear amplification and distinct melting profiles for the wild and mutant alleles of *KLK3, HNF1B, and JAZF1* (Figure 4). A repeat of the HRM analysis of *KLK3*-positive controls showed improved amplification efficiency and enhanced discrimination of wild-type, mutant, and heterozygous genotypes (Figure 4).

### 3.4. Genotype and Allele Frequency Distribution

Based on HRM genotype data (Table 5), the prostate cancer (PCa) cohort (*n* = 13) showed a higher frequency of putative risk alleles at all three investigated loci than the benign prostatic hyperplasia (BPH) cohort (*n* = 12). For rs2735839 *(KLK3)***,** the homozygous variant genotype (GG) was observed in 30.7% of PCa cases compared with 8.3% of BPH controls, while the G allele frequency was 61.5% and 29.2%, respectively. Similarly, for rs4430796 *(HNF1B)***,** the C allele occurred more frequently in PCa participants (53.8%) than in BPH participants (37.5%), and the heterozygous TC genotype was the most common among cancer cases (53.8%). For rs10486567 *(JAZF1)***,** the T allele frequency was higher in the PCa group (57.7%) than in the BPH group (29.2%), and the TT genotype was observed more frequently among cancer cases (30.7%) than BPH controls (8.3%). The genotype and allele association analyses are presented in Table 5. For *KLK3* rs2735839, carriers of the AG and GG genotypes had higher estimated odds of prostate cancer than participants with the AA genotype, although the genotype-specific associations were not statistically significant. Under the allelic model, the G allele was significantly more frequent in the prostate cancer group than in the BPH group and was associated with approximately fourfold higher odds of prostate cancer compared with the A allele (OR = 3.89, 95% CI: 1.19–12.68; Fisher’s exact *p* = 0.027). For *HNF1B* rs4430796, the TC and CC genotypes produced elevated OR estimates relative to the TT genotype, but the confidence intervals were wide and the associations were not statistically significant. The C allele was also more frequent among prostate cancer participants, although its association did not reach statistical significance (OR = 1.94, 95% CI: 0.63–6.02; *p* = 0.272). For *JAZF1* rs10486567, the CT and TT genotypes were associated with increased point estimates of prostate cancer odds compared with the CC genotype, but neither comparison was statistically significant. The T allele showed a borderline association with prostate cancer (OR = 3.31, 95% CI: 1.02–10.72; Fisher’s exact *p* = 0.052). The broad confidence intervals across the genotype and inheritance-model analyses reflect the limited precision resulting from the small pilot cohort. Therefore, these findings are regarded as exploratory and hypothesis-generating rather than confirmatory evidence of genetic association.

Hardy–Weinberg equilibrium analysis showed that genotype distributions in the benign prostatic hyperplasia (BPH) group did not deviate significantly from Hardy–Weinberg expectations for any of the investigated loci. The genotype distributions for *KLK3* rs2735839 (χ^2^ = 0.001, *p* = 0.97), *HNF1B* rs4430796 (χ^2^ = 0.15, *p* = 0.70), and *JAZF1* rs10486567 (χ^2^ = 0.001, *p* = 0.97) were all consistent with Hardy–Weinberg equilibrium, indicating no evidence of significant departure from expected genotype frequencies in the comparison group (Table 6).

### 3.5. Statistical Association Analysis

Because of the limited sample size, Fisher’s exact test was used to compare genotype distributions between prostate cancer and BPH groups. Although the *KLK3* G allele, *HNF1B* C allele and *JAZF1* T allele were observed more frequently among prostate cancer patients, none reached statistical significance after Fisher’s exact testing (all *p* > 0.05) except the *KLK3* G allele. Estimated odds ratios suggested a trend toward increased prostate cancer risk associated with these alleles, although confidence intervals were wide because of the limited cohort size. These findings should therefore be regarded as exploratory rather than confirmatory.

## 4. Discussion

This pilot study demonstrates the applicability of high-resolution melting (HRM)- based single-nucleotide polymorphism (SNP) genotyping as a rapid and affordable molecular approach for evaluating the susceptibility of prostate cancer (PCa) and benign prostatic hyperplasia (BPH) in Nigerian men. The findings also provide preliminary evidence that germline variants within *KLK3*, *HNF1B* and *JAZF1* may complement conventional clinical assessment in differentiating malignant from benign prostate disease, particularly in resource-constrained healthcare settings.

The sociodemographic distribution observed in this study aligns with established epidemiological trends in sub-Saharan Africa. Benign prostatic hyperplasia was most prevalent among men aged 61–70 years, whereas prostate cancer incidence peaked in the 71–80-year age group. This age-related pattern is consistent with previous reports indicating progressive prostate disease risk with advancing age [18]. The predominance of Yoruba ethnicity among patients with both prostate cancer and benign prostatic hyperplasia in this study likely reflects the demographic composition of the study area and recruitment centre rather than indicating ethnicity-specific susceptibility. It aligns with findings from previous Nigerian hospital-based reports [19]. Additionally, the overwhelming proportion of married participants is consistent with the socio-demographic trends documented in other West African studies on prostate diseases [20].

Educational attainment was evenly distributed between primary and tertiary education, with a higher proportion of PCa patients having only primary education. Lower educational status has been associated with delayed health-seeking behavior and late-stage presentation. This aligns with reports made by Iheanacho & Enechukwu [21].

The Clinical History, Lifestyle, and Symptomatology of most of the patients in both groups report no family history of cancer, no prior cancer diagnosis, and no history of sexually transmitted infections (STIs), supporting recent epidemiological findings, while family history is a recognized risk factor, with its absence common among African prostate cancer patients [22]. Urinary tract symptoms also varied between groups: PCa patients reported more pain and urge-related symptoms, whereas BPH patients experienced slower stream, straining, and incomplete emptying. These symptom patterns are consistent with recent clinical reviews, which note that irritative and obstructive symptoms are more pronounced in advanced or malignant prostatic disease [23].

Lifestyle assessment revealed frequent use of herbal supplements and carbonated drinks in both groups, with generally low levels of exercise prior to diagnosis. Some patients continued smoking and alcohol consumption. These lifestyle factors have been increasingly implicated in the risk and progression of prostate diseases in African populations [24]. Although dietary habits, including frequent consumption of sugar-sweetened beverages, have been investigated as potential risk factors for metabolic dysfunction, their relationship with prostate cancer remains inconclusive [25].

Clinically, prostate cancer patients exhibited markedly higher PSA levels, body mass index, systolic blood pressure, and Gleason scores compared with BPH patients. Elevated PSA levels and high Gleason scores observed in this cohort reflect the aggressive disease presentation commonly reported among men of African ancestry [24], often attributed to late diagnosis and limited access to advanced screening tools [26]. These findings reinforce the limitations of PSA-based screening alone and underscore the need for adjunct molecular diagnostics.

The HRM analysis successfully differentiated wild-type, mutant, and heterozygous genotypes for SNPs within the *KLK3* and *HNF1B* genes. Notably, mutant alleles were more frequently detected in prostate cancer samples than in benign cases. The *KLK3* gene, which encodes prostate-specific antigen, plays a central role in prostate physiology and pathology. Genetic alterations in *KLK3* have been associated with altered PSA expression, tumor aggressiveness, and disease progression [27]. The predominance of *KLK3* variants observed in this study supports the continued evaluation of KLK3 as a potential molecular biomarker for prostate malignancy.

Similarly, *HNF1B* variants were more frequently detected in prostate cancer samples. *HNF1B* functions as a transcription factor involved in cellular differentiation and tumor suppression, and its dysregulation has been linked to prostate cancer susceptibility and poor prognosis [28]. The presence of mutant *HNF1B*, predominantly in malignant samples, suggests a possible role in prostate cancer development and progression within this population.

In contrast, SNP analysis of *JAZF1* showed limited discriminatory value in this study. While *JAZF1* variants have been associated with prostate cancer risk in some populations [8], their utility may be population-specific or dependent on larger sample sizes and additional molecular context. This observation further emphasizes the need for population-specific genomic studies rather than relying solely on data generated from European or Asian cohorts.

Post hoc power analysis indicated limited statistical power for detecting modest genetic effects because of the small sample size. Consequently, the absence of statistically significant associations should not be interpreted as evidence of absence of genetic effects but rather reflects the exploratory nature of this pilot investigation.

Optimization of DNA input concentration was a key methodological finding. Improved HRM curve resolution and genotype discrimination were achieved using DNA dilutions of 1:100 and 1:1000, highlighting the importance of template optimization in HRM-based assays. This optimization enhances the reliability of HRM as a diagnostic tool, particularly in laboratories with limited resources.

Although HRM genotyping identified a higher frequency of the *KLK3* G, *HNF1B* C and *JAZF1* T alleles among prostate cancer patients than among BPH patients, these observations should be interpreted cautiously. The small sample size limits statistical precision and increases the likelihood that the observed allele enrichment reflects sampling variation rather than true population-level genetic associations. Nevertheless, the observed trends are consistent with previous genome-wide association studies identifying these loci as prostate cancer susceptibility regions.

Similar observations have been reported for susceptibility polymorphisms beyond prostate cancer. For example, polymorphisms within *PHLPP2* (rs61733127) and *PIK3R1* (rs3730089) have been associated with increased susceptibility to colorectal and breast cancers [29], while the *MIR137* variable number tandem repeat (VNTR) polymorphism rs58335419 has been reported to influence susceptibility to colorectal and gastric cancers [30]. Collectively, these studies support the broader concept that inherited germline polymorphisms contribute to cancer susceptibility across multiple malignancies, although validation in ethnically diverse populations remains essential.

This study has some limitations. The relatively small sample size from a single tertiary hospital may limit the generalizability of the findings, and larger multicentered studies are required to validate the clinical utility of the identified SNPs. Additionally, functional studies were not performed to elucidate the biological mechanisms underlying the observed genetic associations. Additional limitations include the absence of sequencing validation of all clinical samples, the lack of adjustment for potential confounding variables such as age and PSA concentration, and the inability to perform multivariable logistic regression because of the limited number of participants.

Despite these limitations, this study represents one of the first evaluations of HRM-based germline SNP genotyping for prostate cancer susceptibility in Nigerian men using validated synthetic controls. Importantly, the primary objective of the present investigation was not to establish definitive genetic associations but to evaluate the feasibility of HRM as an affordable genotyping platform for prostate cancer susceptibility loci in a Nigerian clinical setting. The successful discrimination of all three SNPs demonstrates the robustness of the assay and provides a foundation for adequately powered multi-centre genetic association studies in African populations.

An additional advantage of HRM is its capacity to screen an entire amplified fragment rather than interrogating only a predefined nucleotide substitution. Consequently, an abnormal melting profile may reflect the presence of the target SNP, another known polymorphism, or a previously uncharacterized sequence variant located within the amplicon. This feature makes HRM useful for variant discovery and preliminary screening. However, HRM does not independently determine the nucleotide position or identity of an unexpected variant. Samples producing melting profiles that do not correspond to validated genotype controls should therefore undergo confirmatory sequencing. In the present study, synthetic allele controls were used to classify the expected genotypes, while sequencing of atypical profiles should form part of future assay validation.

## 5. Conclusions

This pilot study demonstrates the technical feasibility of high-resolution melting-based SNP genotyping for *KLK3* (rs2735839), *HNF1B* (rs4430796), and *JAZF1* (rs10486567) in Nigerian men with prostate cancer and benign prostatic hyperplasia. Although higher frequencies of the putative risk alleles were observed among prostate cancer patients, the limited sample size precludes definitive conclusions regarding genetic susceptibility. Nevertheless, the findings suggest that HRM-based SNP genotyping may represent a rapid, reproducible, and affordable molecular approach that warrants validation in larger multi-centre studies before clinical implementation.

## Figures and Tables

**Table 1:** SNP Selection.

S/N	SNP	Gene	Genomic Variant	Alleles
1	rs2735839	*KLK3* (Kallikrein-Related Peptidase 3): Intron Variant	NG_011653.1:g.11453A>C NG_011653.1:g.11453A>G NG_011653.1:g.11453A>T	A>C, G, T
2	rs4430796	*HNF1B* (Hepatocyte Nuclear Factor 1 Beta: Intron Variant	NG_013019.2:g.12058T>G NG_013019.2:g.12058T>C NG_013019.2:g.12058T>A	T>C, G, A
3	rs10486567	*JAZF1* (Juxtaposed with another Zinc Finger 1): Intron Variant	NG_011499.1:g.248875C>T, NG_011499.1:g.248875C>G	C>T, G

**Table 2:** Primer sequence for KLK3, HNF1B and JAZF1 SNPs.

S/N	SNP	Forward Primer	Reverse Primer
1	rs2735839	GTGAGGGAAAGGGAGAAGATG	GAGTTCAGTGTCCTTCCT CAAC
2	rs4430796	AGGAGCCATTGTCTCCAAAG	GCCTCCTTCTCCTTTCTGAAG
	rs10486567	CTAACACTGTTGGCAAAGCCTC	TCACAGACATGCTTGCTGACTC

**Table 3:** Baseline Characteristics Table. Comparison between Prostate Cancer cohort (*N* = 13) and Benign Prostatic Hyperplasia cohort (*N* = 12).

Characteristic	Prostate Cancer	BPH	*p*-Value
Age (years)	72.5 (69.2, 75.5)	60.5 (57.8, 70.0)	0.007
Total PSA (ng/mL)	18.1 (13.5, 139.0)	5.7 (1.8, 13.8)	0.022
Educational level, *n* (%)			0.456
Primary	5 (35.7)	3 (25.0)	
Secondary	2 (14.3)	0 (0.0)	
OND	1 (7.1)	0 (0.0)	
Tertiary (HND/Univ.)	4 (28.6)	4 (33.3)	
MSc	1 (7.1)	1 (8.3)	
NA	1 (7.1)	2 (16.7)	

Continuous variables are presented as median (Q1, Q3); categorical variables as *N* (%).

**Table 4:** Clinical data of Benign Prostatic Hyperplasia and Prostate Cancer Patients. Parameters include average Body Mass Index (BMI), blood pressure (BP), and Prostate Specific Antigen (PSA) levels. Data were summarized using descriptive statistics (mean ± standard deviation) to compare clinical features between the two patient groups.

Parameters	Diagnosis	
	BPH	PCa
Average Body Mass Index (Kg/m^2^)	22.48 ± 0.08	25.09 ± 0.12
Average B.P. (mmHg)	130/77 ± 10/2.5	143/78 ± 9.35/2.35
Average PSA (ng/mL)	9.16 ± 0.12	79.45 ± 0.23

**Table 5:** Genotype and allele distributions of prostate cancer susceptibility SNPs in prostate cancer and benign prostatic hyperplasia participants. Odds ratios estimate the odds of prostate cancer relative to BPH. For genotype comparisons, the common homozygous genotype was used as the reference category. The analysis is based on 13 prostate cancer participants and 12 BPH participants. Data are presented as number and percentage. OR, odds ratio; CI, confidence interval; PCa, prostate cancer; BPH, benign prostatic hyperplasia. Genotype-specific odds ratios were calculated relative to the homozygous reference genotype. Allelic ORs compare the putative risk allele with the corresponding reference allele. Two-sided Fisher’s exact tests were used because of the small cell frequencies. ORs and 95% CIs are unadjusted. Statistical significance was defined as *p* < 0.05.

SNP/Gene	Genotype	PCa, *n* (%)	BPH, *n* (%)	OR (95% CI)	Fisher’s Exact *p*-Value
rs2735839 *(KLK3)*	AA	3 (23.1)	6 (50.0)	1.00 (Reference)	—
	AG	6 (46.2)	5 (41.7)	2.40 (0.39–14.88)	0.406
	GG	4 (30.8)	1 (8.3)	8.00 (0.60–106.94)	0.266
	Dominant model: AG + GG vs. AA	10/13 (76.9)	6/12 (50.0)	3.33 (0.60–18.55)	0.226
	Recessive model: GG vs. AA + AG	4/13 (30.8)	1/12 (8.3)	4.89 (0.46–51.87)	0.322
	A allele	10/26 (38.5)	17/24 (70.8)	1.00 (Reference)	—
	G allele	16/26 (61.5)	7/24 (29.2)	3.89 (1.19–12.68)	0.027
rs4430796 *(HNF1B)*	TT	2 (15.4)	5 (41.7)	1.00 (Reference)	—
	TC	7 (53.8)	5 (41.7)	3.50 (0.47–25.90)	0.350
	CC	4 (30.8)	2 (16.7)	5.00 (0.47–52.96)	0.286
	Dominant model: TC + CC vs. TT	11/13 (84.6)	7/12 (58.3)	3.93 (0.59–26.20)	0.202
	Recessive model: CC vs. TT + TC	4/13 (30.8)	2/12 (16.7)	2.22 (0.33–15.18)	0.645
	T allele	12/26 (46.2)	15/24 (62.5)	1.00 (Reference)	—
	C allele	14/26 (53.8)	9/24 (37.5)	1.94 (0.63–6.02)	0.272
rs10486567 *(JAZF1)*	CC	3 (23.1)	6 (50.0)	1.00 (Reference)	—
	CT	6 (46.2)	5 (41.7)	2.40 (0.39–14.88)	0.406
	TT	4 (30.8)	1 (8.3)	8.00 (0.60–106.94)	0.266
	Dominant model: CT + TT vs. CC	10/13 (76.9)	6/12 (50.0)	3.33 (0.60–18.55)	0.226
	Recessive model: TT vs. CC + CT	4/13 (30.8)	1/12 (8.3)	4.89 (0.46–51.87)	0.322
	C allele	11/26 (42.3)	17/24 (70.8)	1.00 (Reference)	—
	T allele	15/26 (57.7)	7/24 (29.2)	3.31 (1.02–10.72)	0.052

**Table 6:** Hardy–Weinberg Equilibrium Analysis of the BPH Group. Genotype distributions in the BPH group were evaluated for Hardy–Weinberg equilibrium. No significant deviation from Hardy–Weinberg equilibrium was observed (all *p* > 0.05).

SNP	HWE χ^2^	HWE *p*-Value
rs2735839 (*KLK3*)	0.001	0.97
rs4430796 (*HNF1B*)	0.15	0.70
rs10486567 (*JAZF1*)	0.001	0.97

## Data Availability

The original contributions presented in this study are included in the article. Further inquiries can be directed to the corresponding author.

## Supplementary Materials

The following supporting information can be downloaded at: https://www.mdpi.com/article/10.3390/diagnostics16162521/s1, File S1: Structured study questionnaire.

## Abbreviations

SNP	Single Nucleotide Polymorphism
HRM	High-Resolution Melting
PCR	Polymerase Chain Reaction
PCa	Prostate Cancer
BPH	Benign Prostatic Hyperplasia
LUTH	Lagos University Teaching Hospital
TGL	The GeneLab
DNA	Deoxyribonucleic acid
SPSS	Statistical Package for the Social Sciences
MIQR	Median Inter Quartile Range
CMUL	College of Medicine, University of Lagos
HREC	Health Research Ethics Committee
PSA	Prostate-Specific Antigen
ELISA	Enzyme-Linked Immunosorbent Assay
*KLK3*	Kallikrein-Related Peptidase 3 gene
*HNF1B*	Hepatocyte Nuclear Factor 1 β gene
*JAZF1*	Juxtaposed with Another Zinc Finger Protein 1 gene
WT	Wild-type
MT	Mutant

## Appendix A

**Table A1:** DNA Concentration and purity results for Benign Prostatic Hyperplasia, Prostate Cancer and Healthy control samples. Concentrations are reported in ng/µL, with purity assessed by A260/A280 and A260/A230 ratios.

Benign Prostatic Hyperplasia Samples			
Sample Code	260/280 Ratio	260/230 Ratio	ng/µL
BPH 5	1.86	1.66	32.2
BPH 7	1.96	1.65	28.9
BPH 11	1.77	1.54	17.1
BPH 12	1.82	1.67	20.9
BPH13	1.85	1.50	19.6
BPH 16	1.88	1.23	15.4
BPH 17	1.75	1.51	13.5
BPH 20	1.90	1.23	28.8
BPH 22	1.80	0.89	12.7
BPH 24	1.70	1.59	17.1
BPH 26	1.84	1.64	23.9
BPH 28	1.89	0.97	32.3
**Prostrate Cancer Samples**			
**Sample Code**	**260/280 Ratio**	**260/230 Ratio**	**ng/µL**
PCA 14	1.77	1.69	10.9
PCA 25	1.89	1.75	28.45
PCA 52	1.78	1.79	16.01
PCA 54	1.81	1.68	25.50
PCA 55	1.80	2.11	13.32
PCA 56	1.76	1.66	18.97
PCA 62	1.83	1.53	17.60
PCA 63	1.97	1.94	27.30
PCA 64	1.83	0.76	9.91
PCA 70	1.85	0.89	19.76
PCA 71	1.91	1.26	29.79
PCA 72	1.79	1.54	8.23
PCA 73	1.83	1.62	12.00
**Healthy Control Samples**			
**Sample Code**	**260/280 Ratio**	**260/230 Ratio**	**ng/µL**
Healthy control 1	1.87	1.62	31.5
Healthy control 2	1.84	1.41	22.3
Healthy control 3	1.81	2.08	31.4
Healthy control 4	1.93	1.08	28.8
Healthy control 5	1.78	0.82	26.1
Healthy control 6	1.87	1.09	37.4
