# Supplementary material for: High-Resolution Melting PCR-Based SNP Germline Genotyping of KLK3, JAZF1, and HNF1B in Prostate Cancer and Benign Prostatic Hyperplasia in Nigerian Men—A Pilot Study
Source: Diagnostics (Basel). 2026 Aug 10;16(16):2521. doi: 10.3390/diagnostics16162521 (PMC13510942; doi:10.3390/diagnostics16162521)
Supplement: Supplementary file 1 [file diagnostics-16-02521-s001.zip › diagnostics-4459361-supplementary.pdf]

STRUCTURED STUDY QUESTIONNAIRE

High-Resolution Melting PCR-Based SNP Germline Genotyping of *KLK3*, *JAZF1*, and *HNF1B* in Prostate Cancer and Benign Prostatic Hyperplasia in Nigerian Men—A Pilot Study

Interviewer-administered questionnaire for sociodemographic, clinical, lifestyle, and medical-history data

**Participant study code**		**Date of interview**	
**Recruitment group**	□ PCa □ BPH □ Healthy control	**Hospital/clinic**	LUTH Urology Clinic
**Interviewer initials/code**		**Questionnaire version**	Version 1.0
**Medical record number (coded)**		**Date of sample collection**	

Instructions: Complete using the participant's responses and, where indicated, verified clinical records. Tick one option unless multiple responses are allowed. Do not enter the participant's name. Use "NA" where information is unavailable.

Section A. Eligibility and Consent

No.	Question / Variable	Response options	Response / Code
1	Age 50 years or older	□ Yes □ No	
2	Adult male attending the urology clinic or enrolled as an apparently healthy control	□ Yes □ No	
3	Study information explained in a language understood by the participant	□ Yes □ No	
4	Written informed consent obtained before data and sample collection	□ Yes □ No	
5	Participant willing to provide clinical information and blood sample	□ Yes □ No	

Section B. Sociodemographic and Geographical Information

No.	Question / Variable	Response options	Response / Code
6	Age at enrolment (years)	Record completed years	
7	Date of birth, where available	DD / MM / YYYY	
8	Ethnic group	□ Yoruba □ Igbo □ Hausa/Fulani □ Other: ________	
9	State of origin	Specify	
10	Current state of residence	Specify	
11	Local government area of residence	Specify	
12	Place of residence	□ Urban □ Semi-urban □ Rural	
13	Marital status	□ Single □ Married □ Divorced/Separated □ Widowed	
14	Highest educational level completed	□ None □ Primary □ Secondary □ OND/NCE □ HND/University □ Postgraduate	
15	Current or most recent occupation	Specify	

Section C. Diagnostic and Clinical Information

Complete from the participant's medical record where available.

No.	Question / Variable	Response options	Response / Code
16	Clinical category	□ Prostate cancer □ BPH □ Apparently healthy control	
17	Date of diagnosis	DD / MM / YYYY or year only	
18	Basis of diagnosis (tick all applicable)	□ Clinical assessment □ PSA □ DRE □ Ultrasound □ Histopathology/biopsy □ Other	
19	Total PSA concentration at enrolment	ng/mL	
20	Gleason score (PCa participants only)	Record as reported, e.g., 3+4, 4+4	
21	Histopathological diagnosis/grade, where available	Specify	
22	Body weight	kg	

23	Height	m or cm	
24	Body mass index	kg/m²	
25	Systolic blood pressure	mmHg	
26	Diastolic blood pressure	mmHg	
27	Current prostate-related treatment	□ None □ Medical □ Surgical □ Radiotherapy □ Other: ________	

Section D. Personal and Family Medical History

No.	Question / Variable	Response options	Response / Code
28	Previous diagnosis of any cancer	□ No □ Yes; specify type and year: ________	
29	Family history of cancer in a first- or second-degree relative	□ No □ Yes □ Unknown	
30	If yes, relationship and cancer type	Specify	
31	Known family history of prostate cancer	□ No □ Yes □ Unknown	
32	Presence of any chronic medical condition	□ No □ Yes	
33	Type of comorbidity (tick all applicable)	□ Hypertension □ Diabetes □ Ulcer □ Cardiovascular disease □ Kidney disease □ Other	
34	Current long-term medications	□ No □ Yes; list: ____________________________	

Section E. Urogenital and Infection History

No.	Question / Variable	Response options	Response / Code
35	Previous sexually transmitted infection	□ No □ Yes □ Unknown	
36	Type of sexually transmitted infection, if known	□ Gonorrhoea □ Non-specific urethritis □ Syphilis □ Other: ________	
37	History of genital irritation	□ No □ Yes	
38	History of genital discharge	□ No □ Yes	
39	History of genital sores	□ No □ Yes	
40	History of painful urination	□ No □ Yes	
41	History of infection of the sex organs/urogenital tract	□ No □ Yes □ Unknown	
42	Slow or weak urinary stream	□ No □ Yes	
43	Straining to urinate	□ No □ Yes	
44	Sensation of incomplete bladder emptying	□ No □ Yes	
45	Urinary urgency or increased frequency	□ No □ Yes	
46	Nocturia	□ No □ Yes; number of times/night: ________	

Section F. Lifestyle and Exposure History

No.	Question / Variable	Response options	Response / Code
47	Ever smoked cigarettes or other tobacco products	□ Never □ Former □ Current	
48	If former/current, approximate duration	Years: ________	
49	Alcohol consumption	□ Never □ Former □ Current	
50	Usual alcohol frequency	□ Less than monthly □ Monthly □ Weekly □ Daily	
51	Regular physical exercise before diagnosis/enrolment	□ No □ Yes	
52	Usual exercise frequency	□ <1 day/week □ 1–2 days/week □ ≥3 days/week	
53	Use of herbal medicines or supplements	□ No □ Yes; specify: ____________________	
54	Frequency of carbonated/sugar-sweetened drink consumption	□ Rarely/never □ Monthly □ Weekly □ Daily	
55	Other relevant dietary or occupational exposure reported by participant	Specify	

Section G. Sample Collection and Laboratory Linkage

No.	Question / Variable	Response options	Response / Code
56	Blood sample collected	□ Yes □ No	
57	Sample code	Record coded identifier	
58	Date and time of collection	DD / MM / YYYY; HH:MM	
59	Buffy coat prepared for DNA extraction	□ Yes □ No	
60	Serum prepared for PSA measurement	□ Yes □ No	
61	DNA extraction method	QIAamp DNA Mini Kit	
62	DNA concentration	ng/μL	
63	A260/A280 purity ratio	Record value	
64	A260/A230 purity ratio	Record value	
65	DNA met quality-control requirements for HRM-PCR	□ Yes □ No	

Section H. Data Verification and Completion

No.	Question / Variable	Response options	Response / Code
66	Clinical information verified against medical records where available	□ Yes □ No □ Not applicable	
67	Questionnaire checked for completeness	□ Complete □ Incomplete	
68	Reason for missing information, where applicable	Specify	
69	Interviewer signature/initials	Sign or initial	
70	Date questionnaire completed	DD / MM / YYYY	

Additional Notes

____

____

____

____

____

____

Confidentiality notice: This form must contain only coded study identifiers. It should be stored securely and separately from any document containing personally identifying information.
